# Diagnosis of neglected tropical diseases during and after the COVID-19 pandemic

**DOI:** 10.1371/journal.pntd.0008587

**Published:** 2020-08-14

**Authors:** Dziedzom K. de Souza, Albert Picado, Sylvain Biéler, Sarah Nogaro, Joseph Mathu Ndung’u

**Affiliations:** 1 Neglected Tropical Diseases Programme, Foundation for Innovative New Diagnostics, Geneva, Switzerland; 2 Noguchi Memorial Institute for Medical Research, College of Health Sciences, University of Ghana, Accra, Ghana; Swiss Tropical and Public Health Institute, SWITZERLAND

## Introduction

The occurrence of coronavirus disease 2019 (COVID-19) causing severe pneumonia and death was first reported in December 2019 in Wuhan, China. This was subsequently revealed to be similar to severe acute respiratory syndrome coronavirus and named SARS-CoV-2 [1]. Since December 2019, more than 20.6 million cases have been reported, with over 749,000 deaths [2]. The urgent need to develop diagnostics for COVID-19 has resulted in the already under-resourced development and manufacturing of rapid diagnostic tests (RDTs) and molecular tests for many tropical diseases being placed on hold. In this article, we assess the impact that COVID-19, and the strategies put in place to control the pandemic, may have on neglected tropical diseases (NTDs), and in particular on the diagnostic capacity and needs of those diseases.

## NTD control efforts

NTDs represent a group of 20 diseases that affect more than 1 billion people in 149 countries, with the vast majority in Africa, Asia, and the Americas [3]. They represent ancient diseases of stigma and poverty, primarily affecting the poor, vulnerable, and marginalised people in society, causing significant socioeconomic losses to affected individuals, families, communities, and countries, amounting to billions of dollars every year. Overall, NTDs are responsible for more than 500,000 deaths every year [4]. For the past 20 years, these diseases have been the target of various control or elimination efforts, increasing in number, size, and scope. The NTD movement has evolved and gained momentum, starting from the United Nations Millennium Development Goals, the Berlin meetings in 2003 and 2005, the 2012 London Declaration, the World Health Assembly Resolution of 2013 calling on WHO member states to intensify efforts to address NTDs, to the Sustainable Development Goals in 2015 [5]. These initiatives have sought to bring attention to diseases that for a long time have been overshadowed by more prominent diseases such as HIV/AIDS, tuberculosis (TB), and malaria, with the aim of ‘leaving no one behind’ [6]. On 30 January 2020, more than 350 partners around the world came together to celebrate the first ever World NTD Day to highlight the progress made and take action to beat NTDs ‘for good and for all’. On 9 April 2020, the WHO released the new road map for NTDs 2021–2030, which would have been considered for approval by the Seventy-third World Health Assembly (WHA) [7], were the proceedings not delayed due to the COVID-19 pandemic. The road map lays out the basis for the control and/or elimination of NTDs over the next 10 years, and highlights that ‘effective diagnostics are a prerequisite for reaching the 2030 disease targets, as they are essential key components of NTD programmes, from confirmation of disease to mapping, screening, surveillance, monitoring and evaluation’. The Kigali summit on malaria and NTDs planned on 25 June and intended to launch the NTD road map, set the global agenda and power the progress against NTDs through political and financial commitments, had to be postponed [8]. Furthermore, the devastating impact of COVID-19 may well threaten the achievements and progress envisaged for the coming years, especially the research and development needs for NTD diagnostics, through reduction in funding, human resources, clinical trials, and implementation in the field.

## Funding for NTD diagnostics

Many NTD control and elimination programmes have also been successful due to the availability of diagnostic tools. For example, the Global Programme to Eliminate Lymphatic Filariasis (GPELF) is considered one of the most successful public health programmes, due to the availability of an RDT to map distribution of the disease, and effective drugs for treatment. Since its inception in the year 2000, more than 7.7 billion treatments have been given to over 910 million people [9]. This has led to the significant reduction in prevalence, infection intensity, and morbidity associated with elephantiasis. Fourteen countries have been certified free of the disease, and mass treatment is no longer required in 10 other countries [9]. In many areas of eastern Africa and the Indian subcontinent, the availability of an improved RDT for visceral leishmaniasis [10] has made diagnosis more accessible and safer compared with invasive methods that require spleen biopsies. This has enabled early treatment and improved patient outcomes and passive surveillance towards the control and elimination in endemic countries. Buruli ulcer, the debilitating skin-eating infection, can now be treated more effectively, and the prospects for new RDTs and point of care tests [11] would enhance early diagnosis and improved treatment outcomes for patients, and mapping towards control activities. However, for many NTDs, their control, elimination, and post-elimination surveillance require diagnostics with better performance, and the unmet diagnostic needs and lack of investment represent significant challenges in achieving these goals. The 2019 G-FINDER report [12] indicated a drop in funding for NTD research. While the overall funding for diseases that have a disproportionate burden on poorer countries reached over US$4 billion in 2018, funding for NTDs has decreased by nearly 10% over the past decade. The current investment for NTDs stands at US$341 million or 23 cents per affected individual, and is woefully inadequate. Of this, only a small fraction goes towards research, development, and procurement of RDTs for NTDs, despite their central role in enabling effective diagnosis, individual case management, community-directed treatment, and surveillance. As a result of the COVID-19 pandemic, current indications are that NTD funding is being reduced further in 2020 and 2021 due to financial challenges or to diversion of funding towards control of the pandemic. Already, some funding rounds have been cancelled as a result of COVID-19. For example, the Global Disease Eradication Fund of the Korea International Cooperation Agency recently decided to cancel their entire new funding round that was expected to start in 2021, as their revenue depends exclusively on an air ticket solidarity levy system [13].

The development and manufacture of RDTs is considered a capital intensive venture with many phases, including prototype development and technical validation, manufacturing validation, performance evaluation and clinical validation, and endorsement and scale-up [14]. This represents a particular challenge for NTDs, especially with high product prices, a limited number of manufacturers, and frequent stock-outs, which reduce access to RDTs and their use where needed. A practical example is the procurement of RDTs for visceral leishmaniasis, which is challenging as countries are unable to meet the minimum order quantity established by the producers, coupled with other logistical challenges [15]. RDTs to support human African trypanosomiasis elimination have also been subsidised to enable their access and use [16]. The control of COVID-19 as a global public health effort could lead to even more neglect of NTDs, with the highly under-resourced development and manufacturing of RDTs for some of these diseases being deprioritised to enable the development and production of tests for SARS-CoV-2. Similar challenges have been observed for other diseases that are much better resourced, such as HIV/AIDS, TB, and malaria. It is estimated that the impact of COVID-19 on the diagnosis of these three diseases will worsen their burden [17–19]. For example, modelling from the TB Alliance indicates that it may take years for the TB burden to return to pre-lockdown levels, as restrictions on society due to COVID-19 appear to severely interfere with TB diagnosis and notifications [18]. One can only imagine the COVID-19 impact on NTDs, considering that these are poorly resourced compared with HIV/AIDS, TB and malaria.

## Universal health coverage

The COVID-19 pandemic has also resulted in disruptions in universal health coverage, the basis for ‘leaving no one behind’ in the fight against NTDs. Recently, WHO published an interim guidance on NTDs recommending a suspension of active case search, mass screening, and treatment activities until such a time when the pandemic is under control [20]. The implication of this guideline is that in many countries, key components of current NTD control and elimination strategies, such as active screening for human African trypanosomiasis and mass drug administration (MDA) activities for lymphatic filariasis, soil-transmitted helminthiasis, schistosomiasis, and many other NTDs are on hold, making NTD control and elimination more difficult. While the gains made before the COVID-19 pandemic can cushion the recurrence and rebound in prevalence for many NTDs, the impact of the pandemic on NTDs could be devastating in the long term, if appropriate actions are not taken quickly to address the challenges.

## Opportunities for strengthening of NTD and COVID-19 strategies

The current crisis may also represent opportunities for strengthening specific components of strategies to control NTDs, such as enhancing the capacity for passive screening using RDTs, as is done for human African trypanosomiasis and visceral leishmaniasis [21,22], using existing healthcare infrastructure for mapping preventive chemotherapy diseases and delivering drugs that would otherwise be given in MDA programmes. This approach may, however, not be feasible for NTDs such as rabies and snakebite envenoming, for which such tests are not available.

The capacity of NTD programmes could also be leveraged to convey COVID-19–related messages, interventions, and logistics to rural populations [23]. Experience from NTD programmes in running large-scale field testing, as is done for lymphatic filariasis or soil-transmitted helminthiasis through community or school surveys, could be used to define control strategies for COVID-19. Contact tracing for all patients with laboratory-confirmed COVID-19 has been one of the strategies adopted by ministries of health to interrupt transmission and curb the incidence. Such an approach has been shown to be highly successful for other diseases. For example, early case detection (through contact tracing) and prompt treatment have been the cornerstones in the fight against leprosy [24]. Visceral leishmaniasis elimination programmes in the Indian subcontinent have also relied on identifying people with fever through ‘fever camps’ [25]. Thus, integrating COVID-19 testing into local health services, or piggybacking onto certain NTD programmes that have well-organised testing capabilities, provides a unique opportunity for increasing COVID-19 testing, especially in harder to reach populations.

Smartphone apps and other phone-based technologies are being used to track cases and identify people at risk of SARS-CoV-2 infection [26]. The use of these technologies may not be possible in NTD endemic areas due to the weakness of the internet network and the limited use of smartphones. However, short message service (SMS)-based systems have been used to improve surveillance of NTDs [27], reporting of diagnostic results and follow-up of patients suffering these diseases. Similar systems could be used to report the results of COVID-19 testing in NTD endemic regions.

Finally, the interruption of mass treatment campaigns for NTDs in many countries as a result of the COVID-19 pandemic could provide research opportunities. For example, these interruptions could be used to reassess NTD infection levels prior to resuming mass treatment activities, thus providing data that would otherwise be difficult to obtain, and thereby confirming the extent to which these and other future interruptions may impact on the road map targets as predicted by the NTD modelling consortium [28].

## Conclusions

There is no doubt that the COVID-19 pandemic deserves attention, due to its high public health impact. Nonetheless, the diagnostic and treatment needs for NTDs should not be ignored in these times, as many more lives are likely to be lost and affected through neglect. This is a call for manufacturers producing RDTs to devote some resources towards NTDs in these challenging times, and for NTD stakeholders to raise awareness around continuing diagnostic and treatment needs. Trillions of dollars have been devoted to COVID-19 activities by governments worldwide. Such abilities to mobilise resources should inform the way forward for NTD control and elimination activities.

The current paradigm for COVID-19 is ‘test, test, test’, and diagnostic tools are widely accepted to be key to control of diseases. The NTD community has been advocating for diagnostic tests to be developed for NTDs, yet control programmes have progressed in recent years with few or underperforming diagnostic tests. Since the start of the COVID-19 crisis, more diagnostic tests have been developed for SARS-CoV-2 than for all 20 NTDs in the last 100 years. Once the COVID-19 pandemic has been controlled, the lessons learnt should include implementing the ‘test, test, test’ motto to all diseases ‘so no one is left behind’. The momentum on diagnostics development for COVID-19 should inform the development of new and better tests for NTDs.
